# Effect of conversion from calcineurin inhibitors to everolimus on hepatitis C viremia in adult kidney transplant recipients

**DOI:** 10.1590/2175-8239-JBN-3860

**Published:** 2018-05-14

**Authors:** Larissa Sgaria Pacheco, Valter Duro Garcia, Ronivan Luis Dal Prá, Bruna Doleys Cardoso, Mariana Ferras Rodrigues, Helen Kris Zanetti, Gisele Meinerz, Jorge Neumann, Diego Gnatta, Elizete Keitel

**Affiliations:** 1 Santa Casa de Misericórdia de Porto Alegre, Departamento de Nefrologia e Transplante de Rim, Porto Alegre, RS, Brasil.; 2 Universidade Federal de Ciências da Saúde de Porto Alegre, Programa de Pós-Graduação em Patologia, Porto Alegre, RS, Brasil.

**Keywords:** Immunosuppression, Hepatitis C, Kidney Transplantation, Viral Load, Imunossupressão, Hepatite C, Transplante Renal, Carga Viral

## Abstract

**Introduction:**

Currently, there is no specific immunosuppressive protocol for hepatitis C (HCV)-positive renal transplants recipients. Thus, the aim of this study was to evaluate the conversion effect to everolimus (EVR) on HCV in adult kidney recipients.

**Method:**

This is an exploratory single-center, prospective, randomized, open label controlled trial with renal allograft recipients with HCV-positive serology. Participants were randomized for conversion to EVR or maintenance of calcineurin inhibitors.

**Results:**

Thirty patients were randomized and 28 were followed-up for 12 months (conversion group, Group 1 =15 and control group, Group 2 =13). RT-PCR HCV levels reported in log values were comparable in both groups and among patients in the same group. The statistical analysis showed no interaction effect between time and group (p value G*M= 0.852), overtime intra-groups (p-value M=0.889) and between group (p-value G=0.286). Group 1 showed a higher incidence of dyslipidemia (p=0.03) and proteinuria events (p=0.01), while no difference was observed in the incidence of anemia (p=0.17), new onset of post-transplant diabetes mellitus (p=1.00) or urinary tract infection (p=0.60). The mean eGFR was similar in both groups.

**Conclusion:**

Our study did not show viral load decrease after conversion to EVR with maintenance of antiproliferative therapy.

## RESUMO

**Introdução:**

Atualmente não há um protocolo imunossupressor específico para os receptores de transplantes renais portadores de hepatite C (HCV). Assim, o objetivo deste estudo foi avaliar o efeito da conversão a Everolimo (EVR) na HCV em receptores adultos de transplantes renais.

**Método:**

Trata-se de um estudo unicêntrico, prospectivo, randomizado, exploratório, controlado, aberto em receptores de aloenxertos renais com sorologia positiva para HCV. Os participantes foram randomizados para conversão a EVR ou manutenção dos inibidores da calcineurina.

**Resultados:**

Trinta pacientes foram randomizados e 28 foram acompanhados por um período de 12 meses (grupo de conversão, Grupo 1 = 15 e grupo controle, Grupo 2 =13). Níveis de RT-PCR HCV descritos em valores logarítmicos foram comparáveis entre os grupos e entre pacientes em um mesmo grupo. A análise estatística não mostrou efeitos de interação entre tempo e grupo (valor p G*M= 0,852), ao longo do tempo em cada grupo (valor p M=0,889) e entre grupos (valor p G=0,286). O Grupo 1 apresentou uma maior incidência de eventos de dislipidemia (p=0,03) e proteinúria (p=0,01); não houve diferença na incidência de anemia (p=0,17), diabetes mellitus de início pós-transplante (p=1,00) ou infecção do trato urinário (p=0,60). A TFGe média foi semelhante nos dois grupos.

**Conclusão:**

Nosso estudo não mostrou redução da carga viral após conversão a EVR com manutenção do tratamento antiproliferativo.

## INTRODUCTION

In recent years, chronic hepatitis C virus (HCV) infection has been recognized as an important health problem worldwide.^1^ Research has shown that the prevalence of HCV infection is significantly higher in hemodialysis and kidney transplant recipients than in the general population. A higher HCV prevalence is associated with a history of multiple blood transfusions and long-term hemodialysis, which are commonly required treatments for these patients.^2^

Kidney transplantation alone is considered the treatment of choice for patients with end-stage renal disease (ESRD), preserved liver function, and no liver cirrhosis. However, data on the outcomes of HCV-positive kidney transplant recipients compared to HCV-negative recipients remain contradictory. Some studies report a lower patient survival rate of HCV-positive kidney recipients in comparison to HCV-negative recipients, whereas other studies report similar outcomes between these two groups.^3^^,^ 4

There is a four- to seven-fold increase in HCV viremia after transplantation when compared to the pretransplant period. It has been suggested that the spectrum of immune response to the virus in immunosuppressed patients is as variable as in immunocompetent patients.^5^ Moreover, there is no evidence-based specific regimen of immunosuppressants for HCV-positive recipients. A retrospective study showed that patients treated with tacrolimus have similar hepatitis viral load and rates of liver fibrosis to patients treated with cyclosporine, although renal function was better in patients treated with tacrolimus.^6^

A recent study on liver transplantation showed a beneficial effect of sirolimus (SRL) on viral recurrence monitored by transaminases, viral load, and histological examination. The study also reported improved survival rates after liver transplantation of HCV-positive patients receiving SRL in comparison to patients on calcineurin inhibitor-based regimens.^7^ No significant changes in the logarithm of viral copies nor any alteration of liver function was observed in HCV-positive kidney transplant recipients that switched to SRL, but liver transplant recipients on SRL monotherapy showed a decrease in viral replication.^8^^,^ 9

Similar to SRL, everolimus (EVR) is a potent mTOR inhibitor (mTORi) and has been used as an immunosuppressive agent in kidney transplantation. Until recently, the only HCV treatment available was based on gamma-interferon use, a therapy associated with a high risk of rejection. During this study's development, the results of direct-acting antiviral treatments became available, and these drugs were shown to be safe, effective, and have minimal side effects in kidney transplant recipients.^10^^-^ 13 However, HCV treatment still needs further research; no specific immunosuppression protocol is available for these patients. Moreover, most clinical trials exclude HCV-positive patients. To the best of our knowledge, only one non-randomized study^14^ can be found in the literature concerning the use of mTOR inhibitors as potential drugs for reducing HCV viral load in renal transplantation patients, justifying the importance of the present study.

## MATERIALS AND METHODS

### STUDY DESIGN

This is an exploratory, single-centered, prospective, randomized, open-label, controlled trial aiming to compare the HCV viral load of kidney transplant recipients converted to EVR versus patients maintained on calcineurin inhibitors (CNI). The study's protocol was approved by an independent ethics committee and registered in the ClinicalTrials.gov database, no. NCT01469884. All subjects signed a written informed consent before enrollment; the study was conducted according to the Good Clinical Practices guidelines and to the Declaration of Helsinki. This study was partially funded by Novartis.

### POPULATION

Adult renal transplant recipients with a positive serology for HCV on CNI therapy with at least three months of follow-up were considered for enrollment. Exclusion criteria were (i) recipients of multiple organ transplants, (ii) eGFR < 30 mL/min, (iii) urinary protein/creatinine ratio > 0.5 (g/g), (iv) severe dyslipidemia, (v) human immunodeficiency virus-positive serology, (vi) hepatitis B-positive serology, (vii) hepatic cirrhosis, and (viii) patients with acute rejection episodes during the 3 months before enrollment.

### DATA COLLECTION

Patients were monitored on an outpatient basis and clinical and laboratory data were evaluated every 3 months. Laboratory follow-up included routine laboratory tests, HCV RNA, and the testing of blood levels for each immunosuppressive medication. Clinical adverse events, including drug-related side effects, rejection, infection, and laboratory abnormalities, were documented at each visit.

### QUANTIFICATION OF SERUM HCV RNA

Serum samples were collected prospectively at the study site every 3 months for 12 months after patient enrollment. Serum HCV RNA was quantified by real-time reverse transcription polymerase chain reaction (RT-PCR) analysis (Abbott Real-Time HCV, Abbott Molecular Ind. Des Plaines, IL 60018 USA). Viral load was expressed in log values.

### RANDOMIZATION

Eligible patients were randomized (1:1). A random number sequence was generated by a computer program and placed in sequentially numbered opaque envelopes.

#### TREATMENT ARMS

Group 1: EVR + antiproliferative drug and/or prednisone. The conversion was performed abruptly for all patients. CNI was discontinued one day before the day of conversion (day 1). EVR administration started on day 1 and a 1.5 mg dose was adjusted twice a day to maintain the EVR whole blood trough level between 6 and 10 ng/mL. The antiproliferative drug (mycophenolic acid or azathioprine) was maintained and could not be permanently withdrawn during the study or conversion. The prednisone dose was not changed until adequate levels of EVR were reached, and its withdrawal was not allowed at any time after conversion.

Group 2: CNI + antiproliferative drug and/or prednisone. Patients were maintained on CNI [tacrolimus (TAC), adjusted to maintain a whole blood trough level between 5 and 10 ng/mL or cyclosporine (CyA), adjusted to maintain a whole blood trough level between 100 and 200 ng/mL]. The antiproliferative drug (mycophenolic acid or azathioprine) and prednisone were maintained and could not be permanently withdrawn during the study.

#### DEFINITIONS

Biopsy-confirmed acute rejection episodes were graded according to Banff 2007 classification. Trough level (C0) was used for whole blood concentrations of cyclosporine and tacrolimus. Severe dyslipidemia was considered when fasting triglycerides were ≥ 400 mg/dL or fasting total cholesterol was ≥ 350 mg/dL or LDL-cholesterol was ≥ 160 mg/dL, despite the use of optimal lipid-lowering therapy.

#### PRIMARY END POINT

The primary end point was the decrease of two or more orders of magnitude in HCV viral load of adult kidney recipients after their conversion from CNI to EVR.

#### SECONDARY END POINTS

Secondary end points included treatment failure, graft loss, or death. We also evaluated renal function (eGFR by the MDRD formula) and urine protein/creatinine ratio (g/g). Safety analyses included the incidence of adverse events, such as dyslipidemia, new onset of diabetes mellitus after transplantation, anemia, urinary tract infection, acute rejection, or malignancies.

#### STATISTICAL ANALYSIS

Primary and secondary end points were analyzed in the intention-to-treat population. Treatment groups were compared using the χ^2^ or Fisher's exact test, with qualitative variables presented as numbers and percentages. Quantitative variables verified by the Shapiro-Wilk test (for normal distribution), presented as means and standard deviations, were compared using the *T*-test. The median time after transplantation was compared using the Mann-Whitney U test (interquartile range for non-normal distribution). The primary end points were analyzed using repeated-measures analysis of variance (ANOVA). All statistical tests were two-sided with a 0.05 level of significance and were performed using the SPPSS software version 20.

## RESULTS

### POPULATION

All patients who fulfilled the inclusion criteria were invited to participate in the study. Thirty patients were enrolled between January 26, 2012 and December 16, 2014. They were followed-up for one year after randomization. Twenty-six patients completed the one-year trial period. Two patients were considered as screening failures due to HCV-negative serology. One patient was removed from the study due to proteinuria/creatinuria ratio above one. One patient withdrew consent. All randomized patients received the assigned treatments and were included in the intention-to-treat population.

Eight out of 30 patients received an induction therapy with antithymocyte globulin (ATG) or IL-2 receptor antagonist (IL2RA). The distribution was similar between the two trial groups. The clinical and demographic characteristics of the patients are shown in Table 1.

**Table 1:** Clinical and demographic characteristics of adult kidney transplant recipients at the moment of enrollment.

	Total Patients N = 30	Controls (CNI) N=15	Conversion (EVL) N=15	P
Recipient age, years (mean ± SD)	39.80 ± 11.3	39.67 ± 1.9	40.00 ± 11.1	0.93
Recipient gender, male, N (%)	21 (70.0)	11 (73.3)	10 (66.7)	1.00
Recipient ethnicity, Caucasian, N (%)	25 (83.3)	11 (73.3)	14 (93.3)	0.33
Baseline Condition				
Hypertension (%)	6 (20.0)	4 (26.7)	2 (13.3)	
APKD (%)	2 (6.7)	1 (6.7)	1 (6.7)	
CGN (%)	3 (10.0)	1 (6.7)	2 (13.3)	
Undetermined (%)	11 (36.7)	5 (33.3)	6 (40.0)	
Other (%)	8 (26.7)	4 (26.7)	4 (26.7)	
Panel-reactive antibody levels above zero, N (%)*	16/25 (64)	8 (66.7)	8 (61.5)	1.00
HLA mismatches (mean ± SD)	3.31 ± 1.43	3.20 ± 1.56	3.45 ± 1.29	0.66
Time elapsed since transplantation (med IR), months	84.59 (23.75;129.0)	84.00 (26.00;123.0)	85.00 (19.00;154.0)	0.78
New onset of diabetes mellitus after transplantation, N (%)	3 (10.0)	1 (6.7)	2 (13.3)	0.10
Donor age, years (mean ± SD)	39.80 ± 13.9	42.3 ± 12.1	37.20 ± 15.4	0.32
Expanded criteria donors, N (%)	2 (6.7)	1 (6.7)	1 (6.7)	1.00
Donor gender, male, N (%)	15 (50.0)	6 (40.0)	9 (60.0)	0.46
Donor type, deceased, N (%)	23 (76.7)	11 (73.3)	12 (80.0)	1.00
Induction therapy, N (%)				
ATG	4 (50.0)	2 (50.0)	2 (50.0)	0.40
IL2RA	4 (50.0)	3 (75.0)	1 (25.0)	
Baseline immunosuppression				
TAC + MPA + PRED (%)	18 (60.0)	9 (60.0)	9 (60.0)	
CYA + AZA + PRED (%)	4 (13.3)	0 (0)	4 (13.3)	
CYA + MPA + PRED (%)	7 (23.3)	5 (33.3)	2 (13.3)	
TAC + AZA + PRED (%)	1 (3.3)	1 (6.7)	0 (0)	
Recipient HCV genotype				
Genotype 3	13 (56.5)	10 (71.4)	3 (33.3)	0.07
Genotype 1	10 (43.5)	4 (28.6)	6 (66.7)	
Subgenotype 1a	7 (70.0)	6 (85.7)	1 (14.3)	
Subgetotype 1b	3 (30.0)	2 (66.7)	1 (33.3)	

APKD = adult polycystic kidney disease; CGN = chronic glomerulonephritis; TAC = tacrolimus; MPA = mycophenolic acid; CYA = cyclosporin; AZA = azathioprine; PRED = prednisone; EVL = everolimus; CNI = calcineurin inhibitor; HCV = hepatitis C virus; HLA = human leukocyte antigens; IR= interquartile range; N = number; Med = median;

* panel-reactive antibody screening was performed for only 25 patients. ATG = antithymocyte globulin; IL2RA = IL-2 receptor antagonist.

### PRIMARY END POINTS

**Graph 1:**
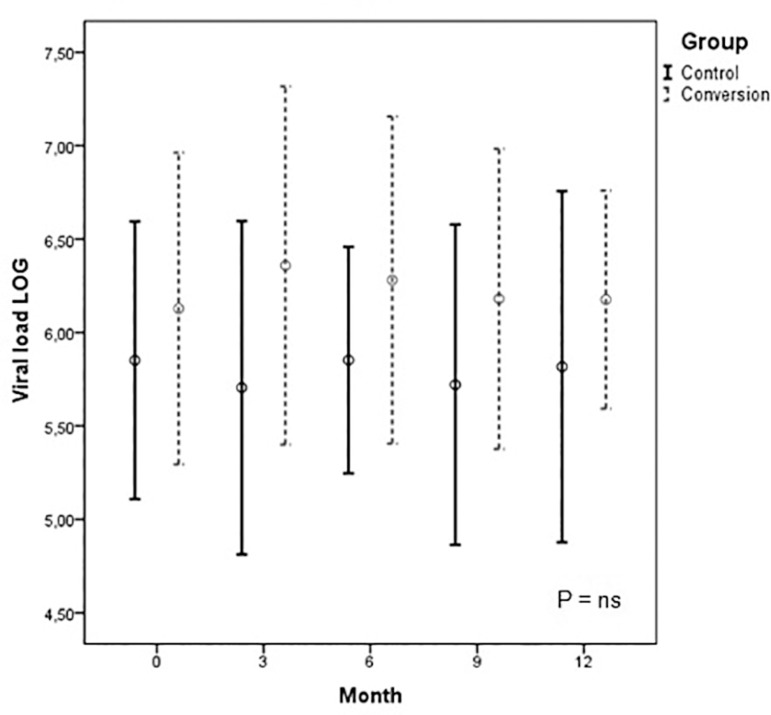
HCV levels, expressed in log values, were comparable between both groups and among patients of the same group. The statistical analysis showed no interaction effect between time and group, between groups over time, and between Group 1 and Group 2.

HCV levels, expressed in log values, were comparable between both groups and among patients of the same group. The statistical analysis showed no interaction effect between time and group (p-value_G*M_ = 0.852), between groups over time (p-value_M_ = 0.889), and between Group 1 and Group 2 (p-value_G_ = 0.286). The mean viral load at baseline, 3, 6, 9, and 12 months were 6.1 ± 0.83, 6.3 ± 0.95, 6.2 ± 0.87, 5.6 ± 1.8, 6.1 ± 0.62, respectively, in Group 1 and 5.8 ± 0.74, 5.7 ± 0.89, 5.8 ± 0.60, 5.7 ± 0.85, 5.8 ± 0.93, respectively, in Group 2 (Graph 1). None of the patients achieved a decrease of two or more orders of magnitude in HCV viral load.

### SECONDARY END POINTS

Patients in Group 1 showed a higher incidence of dyslipidemia (66.7 vs. 23.1%, p = 0.03) and proteinuria events (53.3 vs. 7.7%, p = 0.01) (two patients had p/c ratio > 1.0) when compared to Group 2 (Table 2). During follow-up, there was a reduction in hemoglobin mean in the conversion group (Table 3). One-third of the patients in the conversion group fulfilled the criteria for anemia. However, this difference was not significant when compared to the control group (33.3 vs. 7.7%, p = 0.17). New onset of post-transplant diabetes mellitus (7.7 vs. 6.7%, p = 1.00) and urinary tract infection were similar between the groups (20.0 vs. 7.7%, p = 0.60) (Table 2).

**Table 2:** Adverse events of special interest that occurred during the follow-up period.

	Total Patients N = 28	Control (CNI) N = 13	Conversion (EVL) N = 15	P
Anemia, N (%)	6 (20.0)	1 (7.7)	5 (33.3)	0.17
Dyslipidemia, N (%)	13 (43.3)	3 (23.1)	10 (66.7)	0.03
New onset of post-transplant diabetes mellitus, N (%)	2 (6.7)	1(7.7)	1 (6.7)	1.00
Proteinuria (> 0.5 upr) N (%)	9 (30.0)	1 (7.7)	8 (53.3)	0.01
Urinary tract infection, N (%)	4 (13.3)	1 (7.7)	3 (20.0)	0.60

EVL = everolimus; CNI = calcineurin inhibitor; upr = urine protein/creatinine ratio; N = number.

**Table 3:** Evolution of laboratory test results of both groups during the follow-up period.

	Baseline	Month 1	Month 3	Month 6	Month 9	Month 12	P
AST (U/L)							
CNI	47.76	49.77	40.00*	45.15	41.53	42.30	*0.03
EVL	46.93	57.93	67.33	60.80	58.40	53.14	
ALT (U/L)							
CNI	64.69	60.23	49.54	56.23	54.76	52.00	ns
EVL	47.47	58.27	66.87	60.40	56.80	53.93	
GGT (U/L)							
CNI	102.08	100.38	99.38	104.00	95.30	80.00	ns
EVL	120.20	132.67	146.33	128.13	114.73	105.38	
AP (U/L)							
CNI	80.31	81.08	79.92	89.38	84.72	89.30	ns
EVL	81.40	80.47	82.29	85.87	75.06	72.58	
Hemoglobin (g/dL)							
CNI	15.30*	14.96*	14.87*	15.09*	15.26*	14.93*	*≤0.01
EVL	13.51	12.81	12.50	13.25	13.24	13.05	
Leukocytes (/µL)							
CNI	7264	10834	6295	6887	6833	13888	ns
EVL	5526	5133	5842	5710	5828	5956	
Lymphocytes (/µL)							
CNI	1734	1653	1710	1738	1650	1677	ns
EVL	1524	1446	1449	1650	1616	1547	
Platelets (/µL)							
CNI	182615	177307	186000	188000	176230	185384	ns
EVL	194866	194533	197933	209133	216600	205133	
LDL–C (mg/dL)							
CNI	98.62	96.26*	100.08	98.5	94.30	90.61	*0.02
EVL	99.00	127.21	114.07	114.71	111.20	115.14	
HDL–C (mg/dL)							
CNI	50.69	50.08	48.92	47.08	48.92	46.46	ns
EVL	49.33	46.60	43.20	44.67	46.06	47.64	
Tacrolimus (ng/ml)	8.07	6.60	6.61	5.90	5,78	6.30	ns
Cyclosporine (ng/ml)	51.00	42.66	44.00	44.33	41.33	45.66	ns
Everolimus (ng/ml)	N/A	7.21	5.36	5.02	5.12	4.75	ns

AST = aspartate aminotransferase; ALT = alanine aminotransferase; GGT = gamma-glutamyl transferase;

* Significant difference; ns = not statistically significant (P > 0.05); AP = alkaline phosphatase; EVL = Everolimus; CNI = Calcineurin inhibitor; C = cholesterol.

**Graph 2:**
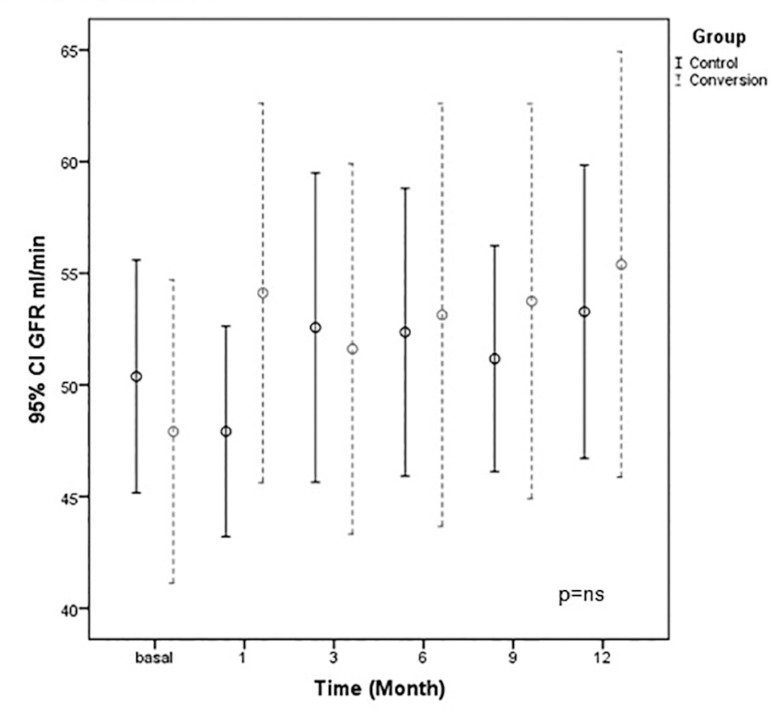
The mean eGFR at baseline, 1, 3, 6, 9, and 12 months after randomization were no statistical difference between the groups.

The mean eGFR at baseline, 1, 3, 6, 9, and 12 months after randomization were 47.91 ± 12.26, 54.12 ± 15.33, 51.08 ± 15.66, 53.13 ± 17.09, 53.74 ± 15.97, 52.99 ± 15.64 mL/min, respectively, in Group 1 and 50.37 ± 8.63, 47.91 ± 7.79, 52.56 ± 11.45, 52.36 ± 10.66, 53.74 ± 15.97, 51.71 ± 9.71 mL/min, respectively, in Group 2. There was no statistical difference between the groups (Graph 2).

Only at the third month of follow-up, aspartate aminotransferase (AST) levels were higher in the conversion group, but this increase was lower than 2.5-fold (Table 3).

The everolimus mean level was kept above 5.0 ng/mL during the follow-up. Only after 12 months of treatment, the levels were slightly lower (4.75 ng/mL), but this difference was not statistically significant (Table 3).

No acute rejection episodes, malignancies, graft losses, or deaths occurred during the follow-up period.

## DISCUSSION

Previous studies and meta-analyses have shown that the use of mTOR inhibitors was associated with lower rates of cytomegalovirus (CMV) infection^15^^,^ 16 and a reduction in the rates of Epstein-Barr virus infection (EBV).^17^^-^ 19 This can be attributed to the limited replication of viruses in biological systems via several pathways and cellular alterations.^20^ The NS5A protein was linked to an increased replication of the hepatitis C virus through p70S6K phosphopeptides. By inhibiting the mTOR/p70S6K pathway, there was a reduction of the phosphorylation of NS5A phosphopeptides *in vivo* and thus a reduction in viral replication.^21^ In addition, the mTOR protein was shown to have a protective role against apoptosis in HCV-infected cells *in vitro*.^22^

In the present study, we could not observe the same results in kidney transplant recipients.^8^ There was no statistical difference in the reduction of viral load between the two groups. None of the patients achieved the expected two-log reduction in viral load during the follow-up period; the results showed that not even a one-log reduction was achieved.

Since only viral load was analyzed but not histological changes in hepatic damage, we cannot be certain of the lack of EVR antiviral activity in HCV-positive patients.

At the third month of follow-up, AST was higher in the conversion group. Intermittent fluctuations of the enzymes may occur as a result of adverse events to various medications or even related to the virus' intrinsic behavior. The correlation between transferase concentration, viral load, and severity of histological lesion is not well established in HCV-positive immunocompetent individuals and renal transplant recipients.^23^

Some studies suggest that the use of mTORi may be associated with a less aggressive evolution of the HCV infection, but the level of evidence is low.^24^ Studies on liver transplantation reported a beneficial effect of mTOR inhibitors on viral load in HCV patients after liver transplantation in comparison to CNI-based regimens.^7^^,^ 9 A retrospective cohort study enrolled 67 HCV-positive recipients of liver transplantation, 39 on mTOR inhibitors and 28 on CNI since the transplant. All patients received a maximum dosage of prednisolone until month 3 and mycophenolate mofetil. Patients in the mTOR inhibitor group showed a decrease of two or more orders of magnitude in viral load between baseline values and months 9 and 12 of follow-up. However, these patients had a viral load at transplant much higher than that observed for patients in the present study.^7^

Soliman et al. performed a prospective non-randomized study and suggested that mTOR inhibitors have the potential to suppress viral replication in HCV-positive renal transplant recipients. Ten patients with allograft dysfunction caused by cyclosporine nephrotoxicity were placed on SRL therapy and compared with 15 patients under cyclosporine (control group). The study showed a significant decrease in HCV PCR levels. However, the study analyzed absolute viral load values instead of log values, different from the recommended by the literature.^25^ The evaluation of absolute values is considered a non-ideal monitoring method due to the high viral load variability detected by RT-PCR.

We believe that EVR whole blood trough levels were not related to the lack of effect in reducing HCV viral load, as the mean level was kept above 5.0 ng/mL. Only in the 12^th^ month of treatment EVR levels were slightly lower (4.75 ng/mL) due to a dose reduction related to side effects. In addition, the time elapsed since transplantation and HCV genotypes were similar between the groups.

There was no available clinical trial data on renal transplants at the time of conception of this trial. This study's main strength is its prospective and randomized nature; its main limitation is the small single-centered nature. A follow-up time of one year is another restriction, although in a study with liver recipients, it was possible to observe different responses in a small number of patients after 9 months of treatment.^7^ The use of mTORi subsequent to transplantation could lead to a more efficient prevention of viral replication; however, in the present study, patients were enrolled in the trial more than 23 months after transplantation.

A study reported that the most prevalent adverse events with mTOR inhibitors were dyslipidemia and proteinuria.^16^ In our study, two patients had a proteinuria/creatinuria ratio above 1. One of them responded to the ACE inhibitor therapy; the other did not respond to this treatment, but proteinuria decreased after conversion to TAC.

Antiviral therapy using interferon and ribavirin were the main approaches to prevent HCV progression until recently, but this therapy is not indicated for renal transplant patients due to the high risk of rejection. During the development of our study, a new generation of direct antiviral agents (DAAs) was approved. The DAAs were shown to be safe and effective, having minimal side effects in kidney transplant recipients.^10^^-^ 13 In Brazil, treatment programs still need further research but show promising results.^26^

In conclusion, our study did not verify a decrease in viral load in HCV-positive renal transplant recipients after conversion to EVR in association with an antiproliferative maintenance therapy.
